# An Interesting Case of Possible Left Atrium Thrombus Versus Fungal Mass in a Patient With Ischemic Cardiomyopathy

**DOI:** 10.7759/cureus.39834

**Published:** 2023-06-01

**Authors:** Mubariz A Hassan, Sinead N Bhagwandeen, Mukarram Jamat Ali

**Affiliations:** 1 Internal Medicine, Howard University Hospital, Washington, D.C., USA

**Keywords:** therapeutic anticoagulation, end stage renal disease (esrd), fungal growth, ischemic dilated cardiomyopathy, left atrial thrombosis

## Abstract

Left atrial masses can present diagnostic challenges due to the wide range of etiologies they can encompass. We present a unique case of a 48-year-old patient with ischemic cardiomyopathy and end-stage renal disease (ESRD) on hemodialysis, who developed a left atrial mass after undergoing intervention with drug-eluting stents. The differential diagnosis included left atrial thrombus versus fungal mass. The patient presented with chest pain and subsequently developed sepsis during the hospital stay, with further workup revealing evidence of fungemia. Transthoracic echocardiography (TTE) demonstrated the presence of a new mass in the left atria. The challenge was to differentiate between a left atrial thrombus and a fungal mass. The patient was managed with a combination of antifungal therapy and anticoagulation and was discharged home.

This case highlights the diagnostic complexities and management considerations associated with left atrial masses in patients with underlying ischemic cardiomyopathy, ESRD, and septic complication versus cardiogenic shock. Accurate differentiation between left atrial thrombus and fungal mass is crucial to guide appropriate treatment strategies. A multidisciplinary approach involving cardiology, infectious diseases, and nephrology is essential in managing such complex cases.

## Introduction

Left atrial masses present a diagnostic challenge in clinical practice, as they can encompass a broad range of etiologies. The occurrence of left atrial masses in the setting of ischemic cardiomyopathy, end-stage renal disease (ESRD), and sepsis poses additional challenges, as these patients are predisposed to both thrombotic and infectious complications. Prompt and accurate diagnosis is crucial to guide appropriate management decisions and optimize patient outcomes.

In this report, we present a unique case of a 48-year-old patient with a past medical history of ischemic cardiomyopathy and ESRD on hemodialysis, who presented with chest pain and underwent intervention with drug-eluting stents. During the hospital stay, the patient developed sepsis, and further investigation revealed evidence of fungemia. Transthoracic echocardiography (TTE) was performed and revealed the development of a new mass in the left atria. The main differential diagnosis considered in this case was a left atrial thrombus versus a fungal mass.

The objective of this case report is to describe the clinical presentation, diagnostic workup, and management approach for this unique case, highlighting the differential diagnosis of left atrial thrombus versus fungal mass. We also discuss the challenges encountered in establishing the diagnosis and the therapeutic strategies employed in this patient, which included a combination of antifungal therapy and anticoagulation. Furthermore, we emphasize the importance of a multidisciplinary approach involving cardiology, infectious diseases, and nephrology in managing such complex cases. This report aims to contribute to the existing literature by presenting a rare presentation of a left atrial mass in a patient with ischemic cardiomyopathy and ESRD. By providing insights into the diagnostic and therapeutic considerations in this context, we hope to enhance the understanding and management of similar cases in the future.

## Case presentation

The case involves a 48-year-old female with a history of coronary artery disease and prior percutaneous coronary intervention (PCI) with a left anterior descending stent placed one year ago (compliant with her dual antiplatelet therapy). She presented with severe, centrally located chest pain, accompanied by vomiting dark brown vomitus, weakness, and fatigue persisting for over a month. Her medical history included ESRD requiring hemodialysis, prior cerebrovascular accident, seizure disorder, esophagitis, hiatal hernia, obstructive sleep apnea, and peripheral artery disease. The patient also denied any current or prior intravenous drug use.

**Figure 1:**
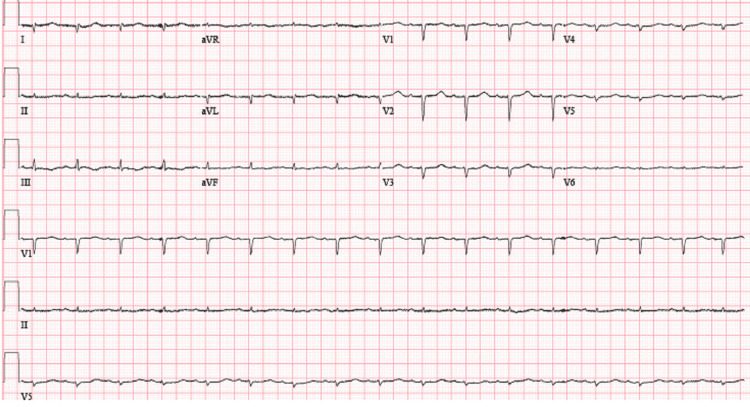
ECG (Electrocardiogram)

Initial assessment revealed hypotension and laboratory findings of elevated creatinine, blood urea nitrogen (BUN), and brain natriuretic peptide (BNP) as shown in Table 1. Additionally, there was a decrease in hemoglobin levels and mildly elevated troponin. A chest X-ray demonstrated cardiomegaly without significant lung abnormalities. The patient was admitted due to concerns about upper gastrointestinal (GI) bleeding and uremic gastritis. Dual antiplatelet therapy was discontinued, and intravenous proton pump inhibitors (PPIs) were initiated. A Nephrology consultation was sought for resuming hemodialysis. Measures such as sequential compression devices (SCDs) were implemented for deep vein thrombosis (DVT) prophylaxis. Electrocardiography (ECG) was done, which was negative for any ischemic changes as shown in Figure 1.

**Table 1:** Laboratory Results

Basic Labs	Results	Reference Range
White Blood Cells	13.13	3.2-10.6x10^9^/L
Hemoglobin	10.7	14.6-17.8 g/dL
Hematocrit	34.5	40.8-51.9 %
Platelets	202	177-406x10^9^/L
Absolute Neutrophils	9.79	1.3-7.1x10^9^/L
Sodium	138	135-145 mEq/L
Chloride	100	95-111 mEq/L
Blood Urea Nitrogen	10	7-25 mg/dL
Creatinine	3.75	0.6-1.2 mg/dL
Potassium	4.6	3.5-5.1 mEq//L
Magnesium	1.94	1.7-2.5 mg/dL
Brain Natriuretic Peptide	4968	<100 Pg/mL
Procalcitonin	0.54	<0.50 ng/mL

Although the patient's chest pain improved after hemodialysis, persistent lethargy and fatigue accompanied by hypotension, tachycardia, and a drop in hemoglobin levels prompted medical optimization. The patient became hypothermic and difficult to arouse, necessitating transfer to the intensive care unit (ICU). Management in the ICU involved addressing the GI bleed, acute encephalopathy, hypoperfusion, and sepsis with empiric antibiotic coverage. Supportive measures, including blood transfusion, intravenous fluid bolus, and vasoactive medications (nor-epinephrine), were administered. A cardiac workup showed an elevation of cardiac enzymes prompting a Cardiology consult for concerns of cardiogenic shock and non-ST-segment elevation myocardial infarction (NSTEMI). The team made the decision to proceed with cardiac catheterization. The procedure involved PCI with stent placement in the left circumflex artery.

**Figure 2:**
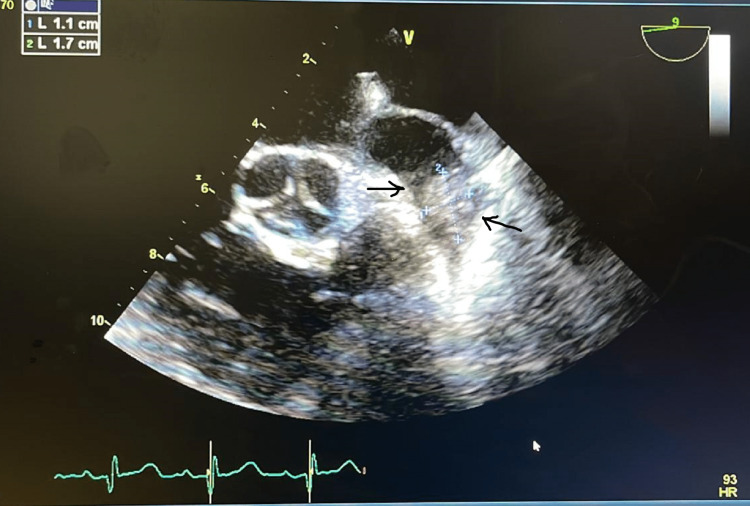
Echocardiogram imaging (apical three-chamber view) showing 3.2x2.0 cm mass in the left atrium (arrows)

Further investigations revealed positive blood cultures for *Candida glabrata*. TTE showed severe global hypokinesis of the left ventricle with an echogenic structure in the left atrium, suggesting a fungal mass versus thrombus as shown in Figure 2. Antifungal therapy with micafungin was initiated along with therapeutic anticoagulation. Following optimized management, the patient experienced improvement and stabilization in her clinical condition. Subsequently, she was discharged from the hospital with arrangements made for outpatient follow-up, including repeat imaging to monitor the progress.

## Discussion

The differentiation between a left atrial thrombus and a fungal mass in the cardiac setting is crucial for accurate diagnosis and appropriate management. Left atrial thrombus formation is commonly associated with conditions such as atrial fibrillation, dilated cardiomyopathy, and myocardial infarction. It poses a significant risk of embolization and subsequent systemic complications. On the other hand, fungal masses in the left atrium are rare but can occur in immunocompromised individuals, particularly those with bloodstream infections. These masses can result from fungal endocarditis or septic emboli and may have distinct clinical implications compared to thrombi.

Imaging techniques play a pivotal role in distinguishing between these entities. TTE is a valuable modality for visualizing intracardiac structures, providing real-time information about size, shape, mobility, and echogenicity. Thrombi typically exhibit layered or mobile morphology, while fungal masses may appear as irregular, friable, or vegetation-like structures. In challenging cases, additional imaging modalities such as cardiac magnetic resonance imaging (CMR) or computed tomography (CT) may be employed. CMR offers superior tissue characterization and can aid in differentiating between thrombi and masses based on their unique signal characteristics [1].

Several case reports provide valuable insights into specific instances of left atrial thrombus and infected thrombi. Prompt diagnosis and appropriate antibiotic therapy to prevent systemic embolization and septic complications are necessary [2]. Therefore, early identification and timely management of large left atrial thrombi to mitigate the risk of adverse events is vital [3]. Distinguishing between atrial masses and thrombi can be challenging. Multimodal imaging techniques, including echocardiography, CT, and CMR, play a crucial role in differentiating between atrial masses of various etiologies and thrombi [4].

Upon literature review, we have also found that prolonged low-dose thrombolytic therapy was used as an adjunctive treatment for an infected right atrial thrombus in some cases. This approach demonstrates the potential benefits of individualized therapeutic strategies in select cases of infected atrial thrombi [5]. Understanding the underlying pathophysiology and morphology of intracardiac thrombi is crucial for accurate diagnosis and appropriate management [6]. Management strategies differ depending on the diagnosis. Anticoagulation therapy is typically indicated for left atrial thrombi to prevent embolic events, while fungal masses require a combination of antifungal therapy and, in some cases, surgical intervention. Prompt identification and accurate differentiation between these entities are crucial for guiding appropriate treatment decisions and optimizing patient outcomes. Therefore, we emphasize the importance of a multidisciplinary approach involving echocardiography, CT, CMR, and clinical assessment for accurate diagnosis and appropriate therapeutic interventions [7].

## Conclusions

We highlight the clinical significance and medical approach of cases with left atrial thrombus and fungal mass. Accurate diagnostic imaging techniques such as echocardiography, CT, and CMR are pivotal in evaluating these conditions. Prompt diagnosis, timely management, and individualized therapeutic strategies are essential to prevent adverse events associated with left atrial thrombus and infected thrombi. Although the management of such complex patients varies from case to case, we believe that combination therapy with antifungal and anticoagulation was a reasonable approach in our case. Hence, we reiterate that multidisciplinary collaboration plays a crucial role in achieving accurate diagnoses and guiding appropriate interventions.
